# HIV-associated tuberculosis: relationship between disease severity and the sensitivity of new sputum-based and urine-based diagnostic assays

**DOI:** 10.1186/1741-7015-11-231

**Published:** 2013-10-29

**Authors:** Stephen D Lawn, Andrew D Kerkhoff, Monica Vogt, Robin Wood

**Affiliations:** 1 The Desmond Tutu HIV Centre, Institute for Infectious Disease and Molecular Medicine, Faculty of Health Sciences, University of Cape Town, Cape Town, South Africa; 2 Department of Clinical Research, Faculty of Infectious and Tropical Diseases, London School of Hygiene and Tropical Medicine, Keppel Street, London WC1E 7HT, UK; 3 George Washington University School of Medicine and Health Sciences, Washington, DC, USA

**Keywords:** Tuberculosis, HIV, Diagnosis, Mortality, Africa, Screening, Xpert, Lipoarabinomannan, Urine

## Abstract

**Background:**

Reducing mortality from HIV-associated tuberculosis (TB) requires diagnostic tools that are rapid and have high sensitivity among patients with poor prognosis. We determined the relationship between disease severity and the sensitivity of new sputum-based and urine-based diagnostic assays.

**Methods:**

Consecutive ambulatory patients enrolling for antiretroviral treatment in South Africa were screened for TB regardless of symptoms using diagnostic assays prospectively applied to sputum (fluorescence smear microscopy, Xpert MTB/RIF and liquid culture (reference standard)) and retrospectively applied to stored urine samples (Determine TB-LAM and Xpert MTB/RIF). Assay sensitivities were calculated stratified according to pre-defined indices of disease severity: CD4 count, symptom intensity, serum C-reactive protein (CRP), hemoglobin concentration and vital status at 90 days.

**Results:**

Sputum culture-positive TB was diagnosed in 15% (89/602) of patients screened and data from 86 patients were analyzed (median CD4 count, 131 cells/μL) including 6 (7%) who died. The sensitivity of sputum microscopy was 26.7% overall and varied relatively little with disease severity. In marked contrast, the sensitivities of urine-based and sputum-based diagnosis using Determine TB-LAM and Xpert MTB/RIF assays were substantially greater in sub-groups with poorer prognosis. Rapid diagnosis from sputum and/or urine samples was possible in >80% of patients in sub-groups with poor prognosis as defined by either CD4 counts <100 cells/μL, advanced symptoms, CRP concentrations >200 mg/L or hemoglobin <8.0 g/dl. Retrospective testing of urine samples with Determine TB-LAM correctly identified all those with TB who died.

**Conclusions:**

The sensitivities of Xpert MTB/RIF and Determine TB-LAM for HIV-associated TB were highest among HIV-infected patients with the most advanced disease and poorest prognostic characteristics. These data provide strong justification for large-scale intervention studies that assess the impact on survival of screening using these new sputum-based and urine-based diagnostic approaches.

## Background

Tuberculosis (TB) remains the leading cause of HIV-related mortality worldwide, accounting for approximately one quarter of HIV/AIDS deaths [1]. A majority of these deaths occur in sub-Sahara Africa, which bears 79% of the global burden of HIV-associated TB [1]. Very high frequencies of undiagnosed disseminated TB have been reported in post-mortem studies of people dying with HIV/AIDS in sub-Saharan Africa both before and during the scale-up of antiretroviral treatment (ART) [2-5]. These data suggest that the true contribution of TB to HIV-related deaths may be underestimated and that failure of ante-mortem diagnosis is a major problem [6].

A number of factors undermine TB diagnosis in people with advanced HIV-related immunodeficiency [7,8]. The clinical presentation is often non-specific, with only a minority of patients with proven pulmonary disease reporting chronic cough of more than two weeks duration [9,10]. Extrapulmonary and disseminated forms of the disease are common and difficult to diagnose [7,8]. In resource-limited settings, heavy reliance is still placed on outdated TB diagnostic tests, such as sputum smear microscopy and chest radiography, both of which have limited diagnostic accuracy in those with advanced immunodeficiency [7,11]. Where culture is available, diagnosis may take several weeks. Thus, TB diagnoses are often either delayed or missed in those with poor immune function and high risk of death.

Over the past few years, considerable progress has been made in the development of new, rapid assays for TB that have useful diagnostic accuracy in patients living with HIV [11-13]. These assays include the Xpert MTB/RIF (Cepheid Inc., Sunnyvale, CA, USA) rapid molecular assay, which can be applied to both respiratory and non-respiratory samples [11]. In addition, the Determine TB-LAM assay (Alere Inc., Waltham, MA, USA) is a simple lateral-flow (strip-test) that detects lipoarabinomannan (LAM) in urine [13]. We have previously reported on the useful diagnostic accuracy of these two assays during active screening of patients prior to starting ART in a South African township [13-15]. However, for new assays to reduce deaths from HIV-associated TB, they must have adequate sensitivity among patients with poor prognosis and highest risk of death. In the present analysis, we therefore ascertained how the sensitivities of urine- and sputum-based approaches to diagnosis using the Xpert MTB/RIF and Determine TB-LAM assays varied according to disease severity as reflected by CD4 cell count, symptom intensity, C-reactive protein (CRP) concentration, hemoglobin concentration and vital status at 90 days of follow-up.

## Methods

The ART services in Gugulethu Township in Cape Town with its high burden of TB and mortality have all previously been described in detail [16-19]. Between 12 March 2010 and 20 April 2011, consecutive patients newly referred to start ART and who were aged >18 years, ART-naive and had no current TB diagnosis were consecutively recruited regardless of symptoms as previously described [13]. All participants provided written informed consent and the study was approved by the research ethics committees of the University of Cape Town, South Africa, and the London School of Hygiene & Tropical Medicine, UK.

Patients were clinically characterized, routine baseline investigations were done and then they were screened for TB. The standardized symptom-screening questionnaire included the World Health Organization (WHO) symptom screen (the presence of more than one of the following symptoms: cough, fever, weight loss or night sweats [9]). Two sputum samples were requested from each patient; a spot specimen was followed by a second that was induced using nebulized 3% hypertonic saline. If necessary, both specimens were induced. Urine samples were collected and stored at −20°C. Blood CD4 cell counts and plasma viral load were measured on all patients via the routine laboratory services. Chest radiographs were obtained and reported by an experienced reader certified in the use of the chest radiograph reading and recording system [20,21].

### Laboratory procedures

Sputum specimens were processed using standardized protocols and external quality assurance procedures by a centralized accredited laboratory as previously described [13]. Samples were decontaminated with N-acetyl-L-cysteine and sodium hydroxide and concentrated by centrifugation. Smears prepared from the sputum pellets were stained with auramine O fluorescent stain for fluorescence microscopy and equal volumes of the remaining pellet were tested by liquid culture and the Xpert MTB/RIF assay. All smears graded as scanty, 1+, 2+ and 3+ were defined as ‘smear-positive’. Cultures were performed using Mycobacterial Growth Indicator Tubes (MGIT, Becton Dickinson, Sparks, MD, USA) and were incubated for up to six weeks. Cultures positive for acid-fast bacilli were identified as *Mycobacterium tuberculosis* complex using the *MTBDRplus* assay (Hain Lifesciences, Nehren, Germany). Xpert MTB/RIF assays were done according to the manufacturer’s instructions. The results of all tests were read by technologists blinded to the outcomes of the other assays.

Frozen urine samples were defrosted and retrospectively analyzed for the presence of lipoarabinomannan (LAM) using the commercially available Determine TB-LAM lateral-flow assay. Positive results were recorded when the test band had equal or greater intensity to the weakest band on the reference card. Defrosted urine samples (2.0 mL) were also concentrated by centrifugation, resuspended in 0.75 mL of phosphate buffer and tested retrospectively using the Xpert MTB/RIF assay.

Concentrations of CRP were measured in duplicate serum samples using the Quantikine enzyme-linked immunosorbent assay (R&D Systems Inc., Minneapolis, MN, USA) according to the manufacturer’s instructions.

### Patient outcomes

Patients were followed up within the routine ART service and patients diagnosed as having TB were referred to treatment clinics within the township. The time to initiation of TB treatment was ascertained and ART service patient records were reviewed to determine vital status at three months.

### Definitions and analysis

Patients were defined as having TB if *M. tuberculosis* was cultured from one or more sputum samples. TB patients were then categorized according to CD4 cell count (>200, 101 to 200 and <200 cells/μL), symptom profile, serum CRP concentration (using strata approximating to quartiles), hemoglobin concentration (using strata corresponding to the WHO classification of anemia [22]) and vital status after three months follow-up, providing stratification by disease severity. Patients were characterized using simple descriptive statistics. The sensitivity of the Xpert MTB/RIF and Determine TB-LAM assays were then calculated for patient groups stratified by the pre-defined indices of disease severity and using culture as the reference standard. Assay sensitivities across these strata were compared using the chi-square and Fisher’s exact tests as appropriate. All statistical tests were two-sided at alpha = 0.05.

## Results

### Patients and TB diagnoses

**Figure 1:**
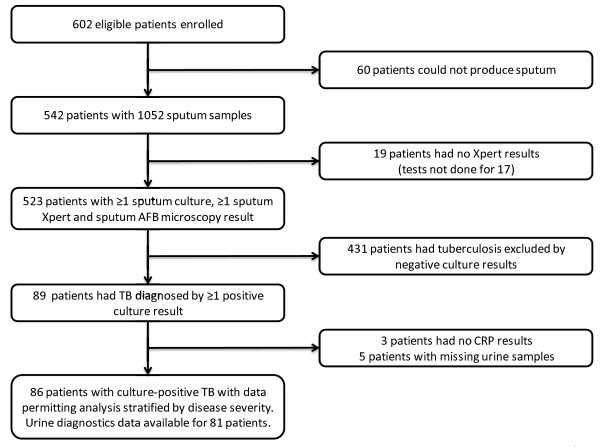
Flow diagram showing the numbers of patients studied.

Of 604 consecutive patients who fulfilled eligibility criteria, 602 agreed to participate. Sputum samples could be obtained from 542 (90.0%) patients. Smear microscopy, culture and Xpert MTB/RIF results were available from one or more sputum samples from 523 patients (Figure 1). Of 89 patients diagnosed with sputum culture-positive TB, data permitting stratification by disease severity were available for 86. Of these, additional data on urine-based diagnostics were available for 81 patients.

Patients with TB (n = 86) had a median age of 33.1 years (IQR, 28.3 to 39.9) and 64% were female. The median CD4 cell count was 131 cells/μL (IQR, 52 to 204) and 47.7% had WHO stage 3 or 4 disease prior to TB screening. The median hemoglobin was 10.8 g/dL (IQR, 8.8 to 12.0) and the median CRP concentration was 57.8 mg/L (IQR, 20.3 to 202.7; range, 1.7 to 560). Symptom severity was classified into three mutually exclusive groups: those with a negative WHO symptom screen (n = 15), those with a positive WHO symptom screen but without cough of two or more weeks’ duration (n = 49) and those with a positive WHO symptom screen including a cough of two or more weeks’ duration (n = 22).

Among the culture-confirmed cases of TB (n = 86), the time to positivity in liquid culture was prolonged (median, 16 days; IQR, 11 to 21). Among 83 patients with evaluable chest radiographs, any radiological abnormality was observed in 61 (73.5%), central abnormalities (mediastinal and hilar lymphadenopathy) were observed in 23 (27.7%), parenchymal abnormalities in 57 (66.3%) and pleural abnormalities in 17 (20.5%). The median number of radiographic zones involved with parenchymal abnormalities was two out of six (IQR, 0 to 4) but was greater in the sub-set of patients with prolonged cough (median, four zones; IQR, 2 to 5).

### Indices of disease severity

Patients with TB (n = 86) were next stratified using four different indices of disease severity: CD4 cell count (<100, 100 to 200 and >200 cells/μL), increasing symptom severity (as defined above), CRP concentration (<20, 20 to 60, 61 to 200 and >200 mg/L) and hemoglobin concentration (<8, 8 to 11 and >11 g/dL). Table 1 shows the characteristics of these groups of patients stratified by these indices and confirms that patients in poorer prognostic groups had other adverse characteristics. In contrast, when patients were grouped according to the presence or absence of chest radiographic abnormalities, it was clear that radiographic appearances in this patient group did not provide a useful index of disease severity (data not shown).

**Table 1:** Characteristics of patients (n = 86) with tuberculosis (TB) stratified by indices of disease severity and survival

	**Blood CD4 cell count**			**Symptom severity**			**C-reactive protein**				**Hemoglobin concentration**			**Vital status at 90 days**	
	**(cells/μL)**						**Concentration (mg/L)**				**(g/dL)**				
	**>200**	**101 to 200**	**0 to 100**	**WHO screen negative**	**WHO screen positive + cough <2 weeks**	**WHO screen positive + cough ≥2 weeks**	**<20**	**20 to 60**	**61 to 200**	**>200**	**>11.0**	**8.0 to 11.0**	**<8.0**	**Alive at 90 days**	**Death at 90 days**
	**(n = 24)**	**(n = 28)**	**(n = 34)**	**(n = 15)**	**(n = 49)**	**(n = 22)**	**(n = 21)**	**(n = 23)**	**(n = 19)**	**(n = 23)**	**(n = 34)**	**(n = 36)**	**(n = 12)**	**(n = 80)**	**(n = 6)**
**Patient characteristics**															
Age, median (IQR)	32.9 (28.6 to 39.0)	31.1 (28.2 to 35.8)	35.5 (25.8 to 44.5)	31.5 (28.6 to 45.9)	33.7 (28.5 to 38.0)	30.5 (26.6 to 39.3)	33.7 (30.7 to 38.0)	35.5 (28.6 to 42.5)	35.2 (28.3 to 44.5)	30.6 (25.5 to 35.6)	35.3 (30.3 to 45.9)	32.9 (26.3 to 40.5)	27.6 (25.0 to 33.1)	32.5 (28.4 to 38.7)	38.6 (26.6 to 44.5)
Female (%)	14 (58.3)	19 (67.9)	22 (64.7)	10 (66.7)	32 (65.3)	13 (59.1)	13 (61.9)	16 (69.6)	13 (68.4)	13 (56.5)	14 (41.2)	29 (80.6)	9 (75.0)	51 (63.8)	4 (66.7)
BMI, median (IQR)	21.2 (20.1 to 24.0)	21.0 (19.7 to 27.4)	21.9 (18.7 to 25.9)	22.6 (19.7 to 27.6)	21.2 (19.3 to 25.0)	21.0 (18.0 to 22.3)	22.0 (20.7 to 27.1)	22.7 (20.9 to 27.6)	20.1 (17.7 to 22.1)	20.6 (18.4 to 24.3)	21.3 (20.6 to 24.5)	21.6 (18.2 to 27.1)	19.8 (17.8 to 24.0)	21.3 (19.6 to 26.0)	18.9 (17.0 to 24.0)
History of previous TB (%)	4 (16.7)	8 (28.6)	5 (14.7)	6 (40.0)	7 (14.3)	4 (18.2)	5 (23.8)	4 (17.4)	2 (10.5)	6 (26.1)	7 (20.6)	8 (22.2)	1 (8.3)	16 (20.0)	1 (16.7)
**WHO stage at enrolment**															
1 or 2 (%)	16 (66.7)	18 (64.3)	11 (32.4)	9 (60.0)	27 (55.1)	9 (40.9)	17 (81.0)	15 (65.2)	4 (21.1)	9 (39.1)	22 (64.7)	16 (44.4)	3 (25.0)	45 (56.3)	0
3 or 4 (%)	8 (33.3)	10 (35.7)	23 (67.7)	6 (40.0)	22 (44.9)	13 (59.1)	4 (19.1)	8 (34.8)	15 (79.0)	14 (60.9)	12 (35.3)	20 (55.6)	9 (75.0)	35 (43.8)	6 (100)
**Blood Tests**															
Hemoglobin g/dL (IQR)	11.5 (10.7 to 13.7)	10.5 (8.5 to 12.7)	9.4 (8.0 to 11.0)	11.5 (10.6 to 13.4)	10.9 (8.9 to 12.0)	8.8 (8.0 to 11.0)	11.7 (11.3 to 14.1)	11.1 (8.8 to 12.3)	10.1 (7.9 to 11.6)	9.0 (7.9 to 10.0)	12.5 (11.7 to 13.9)	9.5 (8.8 to 10.6)	7.3 (7.0 to 7.6)	10.9 (8.8 to 12.3)	9.9 (7.2 to 10.1)
Absolute neutrophil count cells/μL (IQR)	3.3 (2.5 to 4.7)	3.2 (2.4 to 4.3)	4.0 (2.5 to 6.7)	2.6 (1.9 to 3.5)	3.3 (2.4 to 5.3)	4.7 (3.0 to 7.0)	2.5 (1.9 to 3.3)	3.2 (2.5 to 5.0)	5.0 (3.4 to 6.7)	4.3 (2.6 to 9.1)	2.8 (2.0 to 3.5)	3.6 (2 .8 to 5.1)	7.2 (4.4 to 9.4)	3.3 (2.4 to 5.2)	4.9 (4.0 to 6.2)
Absolute lymphocyte count, cells/μL (IQR)	1.9 (1.6 to 2.2)	1.9 (1.4 to 2.7)	0.8 (0.6 to 1.3)	1.9 (1.3 to 2.7)	1.6 (1.1 to 2.0)	1.1 (0.7 to 1.7)	1.6 (1.4 to 2.7)	1.7 (1.0 to 2.2)	1.3 (0.6 to 1.7)	1.4 (0.7 to 2.1)	1.6 (1.3 to 2.3)	1.5 (0.9 to 2.0)	0.5 (0.4 to 1.2)	1.6 (1.1 to 2.1)	0.7 (0.6 to 0.7)
**CD4 cell counts (cells/μl)**															
Median (IQR)	233 (211 to 275)	143 (124 to 184)	35 (21 to 68)	158 (44 to 189)	137 (68 to 213)	109 (35 to 184)	188 (138 to 235)	158 (71 to 205)	52 (21 to 205)	102 (29 to 140)	189 (114 to 235)	98 (41 to 194)	28 (16 to 107)	139 (66 to 205)	34 (29 to 62)
CD4 0 to 100	-	-	34 (100)	5 (33.3)	19 (38.8)	10 (45.5)	3 (14.3)	8 (34.8)	12 (63.2)	11 (47.8)	8 (23.5)	18 (50.0)	8 (66.7)	29 (36.3)	5 (83.3)
CD4 101 to 200	-	28 (100)	-	7 (46.7)	13 (26.5)	8 (36.4)	8 (38.1)	8 (34.8)	1 (5.3)	11 (47.8)	11 (32.4)	9 (25.0)	4 (33.3)	27 (33.8)	1 (16.7)
CD4 >200	24(100)	-	-	3 (20.0)	17 (34.7)	4 (18.2)	10 (47.6)	7 (30.4)	6 (31.6)	1 (4.4)	15 (44.1)	9 (25.0)	0	24 (30.0)	0
**Log viral load (copies/ml)**															
Median (IQR)	4.7 (4.2 to 5.0)	4.6 (4.2 to 5.3)	5.1 (4.7 to 5.5)	4.7 (4.2 to 5.0)	4.8 (4.4 to 5.3)	4.9 (4.5 to 5.4)	4.7 (4.4 to 4.8)	4.7 (4.4 to 5.2)	4.9 (4.5 to 5.6)	5.2 (4.7 to 5.6)	4.4 (3.9 to 4.7)	5.1 (4.8 to 5.4)	5.6 (5.1 to 5.7)	4.8 (4.4 to 5.3)	5.5 (4.9 to 5.7)

### Sensitivity of TB diagnostics and disease severity

**Figure 2:**
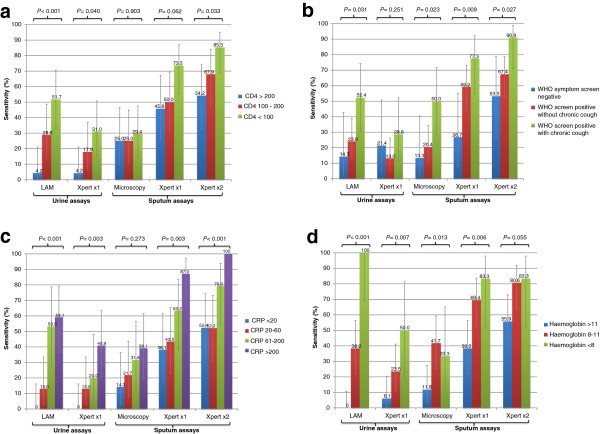
**The sensitivities (%) with 95% confidence intervals of urine-based (n = 81) and sputum-based (n = 86) diagnostic tests for tuberculosis (TB).** Data are shown stratified according to: **(a)** symptoms, **(b)** C-reactive protein (CRP) concentration (mg/L), **(c)** blood hemoglobin concentration (g/dl), **(d)** blood CD4 cell count (cells/μL).

The diagnostic sensitivities of the various sputum-based and urine-based assays differed substantially. The overall sensitivities ranked in descending order were 70.9% (95% CI, 60.1 to 80.2), 58.1% (95% CI, 47.0 to 68.7), 29.6% (95% CI, 20.0 to 40.8), 26.7% (95% CI, 17.8 to 37.4) and 18.5% (95% CI, 10.8 to 28.7) when using Xpert MTB/RIF (two sputum samples), Xpert MTB/RIF (one sputum sample), sputum smear microscopy, Determine TB-LAM (urine) and Xpert MTB/RIF (urine), respectively. However, the sensitivities of these assays varied substantially according to disease severity (Figure 2).

The predominant pattern that emerged from these analyses was that the sensitivities of both urine-based and sputum-based assays tended to be higher among those with lower CD4 cell counts (Figure 2a), more advanced symptoms (Figure 2b), higher CRP concentrations (Figure 2c) and lower hemoglobin concentrations (Figure 2d). These data indicate that rapid diagnosis from sputum and/or urine samples using Xpert MTB/RIF and/or Determine TB-LAM was possible in >80% of patients categorized in the four groups with worst prognosis (CD4 count <100 cells/μL, advanced symptoms, CRP ≥200 mg/L and hemoglobin <8.0 g/dl).

The association between disease severity and diagnostic sensitivity was weakest for sputum smear microscopy for which there was no significant association with CD4 count (Figure 2a) or CRP concentration (Figure 2c). The sensitivity of smear microscopy did not exceed 50.0% in any of the patient sub-groups. In contrast, the sensitivity of Xpert MTB/RIF when testing either one or two sputum samples was substantially greater when comparing the most favorable and least favorable prognostic sub-groups for all of the four indices of disease severity. The median increments in sensitivity were 47.0% (range 27.7 to 50.6) when testing one sputum sample and 34.4% (range, 27.4 to 47.6) when testing two samples.

Although the overall sensitivity of Determine TB-LAM was low, the associations between the sensitivity of Determine TB-LAM point-of-care assay for urine lipoarabinomannan and disease severity were extremely striking (Figure 2). In all of the least favorable prognostic categories, Determine TB-LAM detected a majority of cases, with sensitivity ranging from 51.7% to 100%. When comparing the most favorable and least favorable prognostic categories for each of the four indices of disease severity, the median increment in sensitivity was 53.3% (range, 38.1 to 100.0).

The association between the sensitivity of Xpert MTB/RIF when testing urine samples and disease severity was strong for each of the indices with the exception of symptom severity (Figure 2). In the least favorable prognostic categories, sensitivities ranged from 28.6% to 50.0%. When comparing the most favorable and least favorable prognostic categories for each of the four indices of disease severity, the median increment in sensitivity was 33.9% (range, 7.2 to 43.9).

### Vital status during follow-up and sensitivity of TB diagnostics

**Figure 3:**
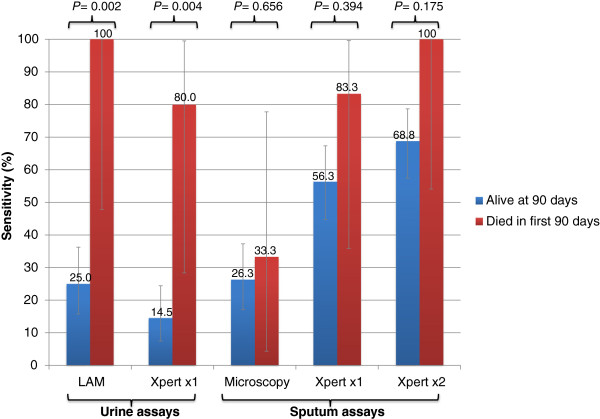
**The sensitivities (%) with 95% confidence intervals of urine-based (n = 81) and sputum-based (n = 86) diagnostic tests for tuberculosis (TB).** Data are shown stratified according to vital status at 90 days.

We next assessed how the diagnostic sensitivity of the assays differed according to vital status at 90 days of follow-up (Figure 3). There was no significant association between the sensitivity of sputum tests (smear microscopy and Xpert MTB/RIF) and vital status, although the sensitivity of Xpert MTB/RIF tended to be higher among those who died. In contrast, despite limited statistical power, there was a very strong relationship between the sensitivity of urine-based tests and vital status at 90 days (Figure 3). The sensitivities of Determine TB-LAM and of Xpert MTB/RIF testing of urine were 75.0% and 65.5% higher, respectively, among patients who died compared to those who survived.

**Figure 4:**
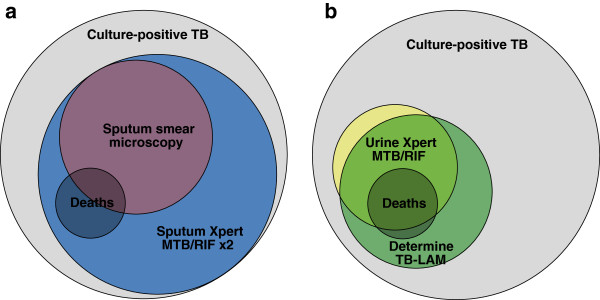
**Venn diagrams showing total tuberculosis cases, deaths and the proportions diagnosed by different diagnostic assays.** Data are shown for 81 cases with complete data using: **(a)** sputum-based investigation with smear microscopy or Xpert MTB/RIF testing of two sputum samples and **(b)** testing urine samples with either Determine TB-LAM or Xpert MTB/RIF.

We next plotted Venn diagrams to reveal the relationship between the sensitivities of the different diagnostic approaches for culture-positive TB cases and for the sub-set who had died during 90 days of follow-up (Figure 4). In contrast to sputum smear microscopy, Xpert MTB/RIF testing of two sputum samples or testing of urine using Determine TB-LAM was able to diagnose TB among all those patients who subsequently died.

## Discussion

Since the advent of the AIDS pandemic, the lack of rapid and accurate assays for HIV-associated TB in those with advanced immunodeficiency has been a major hindrance to reducing deaths from these diseases in resource-limited settings [6]. We found a very high prevalence of culture-positive TB, but diagnosis with conventional tests was challenging. Only one quarter of cases were sputum smear-positive, 26% had normal chest radiographs and the median time to culture positivity was 16 days. However, the key finding of this study was that compared to smear microscopy, the incremental diagnostic sensitivity provided by the Xpert MTB/RIF assay (applied to sputum or urine samples) and the Determine TB-LAM urine test were greatest among the patients with the worst prognostic characteristics: those with the CD4 cell counts <100 cells/μL, advanced symptoms, CRP concentrations >200 mg/L or severe anemia (<8.0 g/dL) and in those who subsequently died. These data indicate that screening sputum and/or urine and samples from patients using these new diagnostic approaches may enable rapid (same-day) diagnosis and treatment in more than 80% of those patients with the worst prognostic characteristics. Use of these approaches therefore has the potential to improve survival and these data provide the basis to justify large-scale intervention trials.

We chose to study four different indices of disease severity that reflected not only the degree of HIV-associated immunodeficiency (CD4 cell counts) but also other important aspects of the disease process. Patients with a negative WHO symptom screen [9] represent those with early ‘sub-clinical’ disease which tends to progress to symptomatic disease over time, presumably as mycobacterial load rises [23,24]. In contrast, those with chronic cough of two or more weeks’ duration had more extensive pulmonary radiographic disease. C-reactive protein is an acute phase protein that reflects the systemic inflammatory response to infection and has prognostic value in patients with HIV-associated TB in this cohort [25]. Anemia is common in patients with HIV-associated TB, especially disseminated disease, and is an independent predictor of mortality in ART programs in sub-Saharan Africa [26-28].

The diagnostic sensitivity of Xpert MTB/RIF testing of sputum was strongly associated with greater disease severity across all prognostic indices. In the sickest patients with the lowest CD4 cell counts, highest CRP concentrations or severe anemia, the sensitivity of a single Xpert MTB/RIF test was more than twice that of smear microscopy. Although high sensitivity of Xpert MTB/RIF was found among those who subsequently died, failure of linkage of patients with the results of tests done in centralized National Health Laboratories Service laboratories and delays in patients returning to the clinic following recall clearly may have undermined any potential impact on survival [29,30]. This illustrates the huge need for rapid point-of-care diagnosis.

The observed higher sensitivity of Xpert MTB/RIF for sputum-based TB diagnosis among those with the lowest CD4 cell counts appears to run contrary to the widely-held dogma that sputum mycobacterial load declines linearly with advancing immunodeficiency. However, the existing view-point is not well supported in the literature. We have recently reported on over 13,000 unselected cases of HIV-associated TB from Cape Town, South Africa [31]. Although the proportion of pulmonary cases testing sputum smear-positive initially declined in parallel with lower CD4 cell counts in the range 500 to 200 cells/μL, the overall relationship was non-linear with the proportion testing smear-positive increasing as CD4 counts declined further from 150 to zero cells/μL [31]. The patterns observed in the present study are entirely consistent with this and suggest that at progressively lower CD4 cell counts below 150 cells/μL, the overall mycobacterial load rises substantially, facilitating microbiological diagnosis when using appropriate clinical samples and diagnostic tools.

Sputum samples were obtained by a dedicated study nurse with the assistance of nebulized hypertonic saline as described in detail elsewhere [32]. The quality of sputum samples is likely to be an important determinant of the yield of TB diagnoses from sputum-based diagnostics and is often much more difficult to obtain under routine program conditions and especially among very sick in-patient populations. A considerable advantage of urine-based TB diagnosis is the ease and rapidity of obtaining and handling urine samples and fewer concerns about sample quality. Urine-based diagnosis, therefore, offers a very important alternative diagnostic approach [33].

Determine TB-LAM is a simple, low-cost lateral flow test that is able to diagnose TB within 30 minutes and provides a rapid means of screening for HIV-associated TB with moderate sensitivity and high specificity among those with the lowest CD4 cell counts [13,34]. This study extends our previous observations to show that although the overall sensitivity of Determine TB-LAM was low when screening unselected HIV-infected patients, useful sensitivity was observed among subsets of patients with poor prognostic features. Thus, the assay might best be used to screen in a targeted fashion HIV-infected patients with low CD4 counts and those with other poor prognostic characteristics such as moderate or severe anemia.

The association of the Determine TB-LAM assay sensitivity with blood hemoglobin concentration was particularly striking, detecting none of the TB cases who had a blood hemoglobin of >11.0 g/dl but detecting all cases with a hemoglobin of <8 g/dl. It is likely that LAM antigenuria reflects disseminated mycobacteremia [35] and possible bone marrow involvement with TB. When retrospectively testing urine samples, Determine TB-LAM correctly diagnosed TB from a single clinical sample in all those who died during the three months follow-up. This assay offers the fastest means of screening for HIV-associated TB, permitting initiation of treatment in the sickest patients pending the results of further investigations.

The Xpert MTB/RIF assay may be used to test a wide range of non-respiratory samples [11]. When testing small volume (2.0 ml) urine samples, the diagnostic sensitivity was not only greater among those with lower CD4 counts as previously reported [15], but was also higher among those with higher CRP concentrations, severe anemia and in those who later died. Xpert MTB/RIF detects DNA from whole *Mycobacterium tuberculosis* bacilli and so all patients with sputum culture-positive TB who also tested urine Xpert MTB/RIF-positive, therefore, had disseminated TB. Sensitivity is increased by concentrating larger volumes of urine by centrifugation [36] and this adds little to the laboratory processing time.

The finding of assay sensitivity varying substantially according to symptom status and disease severity may explain some of the heterogeneity between the results of studies assessing the diagnostic accuracy of tests for TB. This may well reflect differences between study populations. This might explain why, for example, the reported sensitivity of Xpert MTB/RIF for pulmonary TB was substantially lower during community-based active case finding compared to that observed during investigation of sick patients requiring hospital admission (62.6% versus 86.1%, respectively) [37,38]. Thus, studies of the accuracy of TB diagnostic assays should characterize symptom profiles and disease severity of participants.

Strengths of this study include the use of multiple indices of disease severity; the use of sputum induction to obtain high quality sputum samples; access to rigorous quality-assured microbiology laboratories with use of liquid culture as the diagnostic reference standard; the study of both sputum-based and urine-based diagnostic approaches and the ascertainment of vital status at 90 days. The cohort size and numbers of deaths, although limited, were nevertheless sufficient to address the study hypothesis. Weaknesses include the fact that this is a single study site; TB status was ascertained at one time-point and included only patients with sputum culture-positive disease; urine-based assays were done retrospectively and did not inform treatment decisions; and only small volumes of urine were available for Xpert MTB/RIF testing, potentially limiting the yield obtained by this means. We have simply reported on factors affecting diagnostic sensitivity as we have already reported on the specificities of these assays in this cohort which all exceed 98% [13-15]. This study was observational and it cannot be deduced whether use of these new diagnostic approaches would be associated with improved survival.

## Conclusion

In conclusion, sputum-based and urine-based TB diagnosis using Xpert MTB/RIF and Determine TB-LAM assays permit rapid diagnosis of HIV-associated TB among patients with advanced immunodeficiency and especially among those with poor prognostic characteristics. These data provide a strong rationale for large-scale studies of the impact of the use of these assays on survival and on the operational and economic feasibility and sustainability of these approaches.

## Abbreviations

ART: Antiretroviral treatment; CRP: C-reactive protein; LAM: Lipoarabinomannan; MGIT: Mycobacterial growth indicator tubes; MTB/RIF: Mycobacterium tuberculosis/resistance to rifampicin; TB: Tuberculosis.

## Competing interests

The authors declare that they have no competing interests.

## Authors’ contributions

SDL initiated and planned the study. SDL, RW and MV collected the data. SDL and MV ran laboratory assays. ADK did the data analysis. SDL wrote the paper with input from RW and ADK. All authors approved the final version of the manuscript prior to submission.

## Pre-publication history

The pre-publication history for this paper can be accessed here:

http://www.biomedcentral.com/1741-7015/11/231/prepub
